# Identification of NAC Transcription Factors in *Suaeda glauca* and Their Responses to Salt Stress

**DOI:** 10.3390/cimb46080516

**Published:** 2024-08-10

**Authors:** Xujun Fu, Longmin Zhu, Xiaomin Yu, Qinghua Yang, Fengjie Yuan, Hangxia Jin

**Affiliations:** Institute of Crop and Nuclear Technology Utilization, Zhejiang Academy of Agricultural Sciences, Hangzhou 310021, China; fuxj@mail.zaas.ac.cn (X.F.); zlmsllzly@163.com (L.Z.); yuxm@mail.zaas.ac.cn (X.Y.); tsingyang2009@163.com (Q.Y.); fjyuanhz@126.com (F.Y.)

**Keywords:** *Suaeda glauca*, NAC transcription factor, transcriptomics, expression profile

## Abstract

NAC (NAM/ATAF1/2/CUC2) transcription factors regulate plant growth and development and stress responses. Because NAC transcription factors are known to play important roles in the regulation of salt tolerance in many plants, we aimed to explore their roles in the halophyte *Suaeda glauca*. Based on transcriptome sequencing data, we identified 25 *NAC* transcription factor gene family members. In a phylogenetic tree analysis with *Arabidopsis thaliana* NAC transcription factors, the SgNACs were divided into 10 groups. The physicochemical properties and conserved domains of the putative proteins, as well as the transcript profiles of their encoding genes, were determined for the 25 *SgNAC* genes using bioinformatic methods. Most of the *S. glauca NAC* genes were upregulated to some extent after 24 h of salt stress, suggesting that they play an important role in regulating the salt tolerance of *S. glauca*. These findings lay the foundation for further research on the functions and mechanisms of the *NAC* gene family in *S. glauca*.

## 1. Introduction

Stresses such as salinity and drought can reduce the yields of various crops. Plants have evolved a series of regulatory networks to cope with abiotic stresses. Transcription factors are DNA-binding proteins that regulate the expression of multiple genes in a variety of signaling pathways in plants and are involved in various processes such as stress responses, growth and development, and morphogenesis. In many plant species, NAC transcription factors play important roles in the regulation of salt tolerance and plant development. The name of NAC transcription factors is derived from the first letters of *Petunia hybrida* NAM, *Arabidopsis thaliana* ATAF1/2, and CUC2. The N-terminal regions of the *P. hybrida* NAM, *A. thaliana* ATAF1/2, and CUC2 contain a highly conserved amino acid sequence known as the NAC domain. Proteins containing the NAC domain are NAC transcription factors, and they are a unique and important transcription factor family in plants. Members of this family are involved in regulating various biological processes, especially responses to abiotic stresses such as salinity, drought, and low temperature [1,2,3,4,5,6]. For example, *A. thaliana* AtAF2 (ANAC081) is involved in regulating stress resistance, and it acts as a central regulator of plant defense [7,8]. Cotton (*Gossypium* spp.) GhirNAC2 is involved in the response to drought stress. The downregulation of *GhirNAC2* in transgenic cotton lines led to increased stomatal opening and water loss, ultimately reducing tolerance to drought stress [9]. Overexpression of wheat (*Triticum aestivum*) *TaNAC2* in *A. thaliana* enhanced its tolerance to drought, salt, and freezing stress [10].

*Suaeda glauca*, an annual herbaceous plant, is a succulent halophyte belonging to the *Chenopodiaceae* family. This plant is widely distributed in the coastal areas of China. In China, it is not only consumed as a wild vegetable and a medicinal herb but also fed to livestock as a highly nutritious feed. There are no salt glands or vesicles on the leaves of *S. glauca*, but it exhibits strong resistance to salt and alkali stresses and grows well even when the salt content is higher than 0.48%. Under salt stress, *S. glauca* accumulates organic acids and inorganic anions to maintain its intracellular ion balance and stores excess Na^+^ in the vacuoles of mesophyll cells [11,12,13,14,15]. This plant has high salt tolerance, so it is an excellent resource to study the mechanism of salinity tolerance. It may also be a valuable source of genes related to salinity tolerance. Previously, we conducted a transcriptome analysis of salt-treated *S. glauca* and obtained a number of functional candidate genes that respond to salt stress [12]. The results of that study provided the basis for studying the salt tolerance mechanism of *S. glauca*. A systematic study of *NAC* genes in *S. glauca* will provide valuable reference data for functional genomics studies, as well as for molecular breeding.

With the development of high-throughput sequencing technology and the publication of plant genome sequences, more and more *NAC* genes have been discovered. For example, 117 and 151 *NAC* genes have been identified in *A. thaliana* and rice (*Oryza sativa*), respectively [4,16]. However, the NAC family has not been systematically identified and studied in some plants because of the lack of a high-quality reference genome. This study is the first systematic investigation of the conserved domains and phylogenetic relationships of the *NAC* gene family in the halophyte *S. glauca* based on transcriptome data. In addition, the transcript profiles of *NAC* genes under salt stress were determined to identify those involved in the salt stress response. The results of this study provide a theoretical basis for further research on the function of NAC transcription factors in *S. glauca* and their genetic applications.

## 2. Materials and Methods

### 2.1. Identification and Physicochemical Characterization of NAC Family Members in S. glauca

There is no reference genome for *S. glauca*. Therefore, we performed an in silico search for sequences homologous to *NAC* genes in the de novo assembly of the transcriptome of *S. glauca*, which we previously sequenced and uploaded to the SRA database (accession number PRJNA295637). To carry this out, seedlings of *S. glauca* were treated with 300 mmol·L^–1^ NaCl or water as the control. After 24 h, the leaves were frozen in liquid nitrogen, and then RNA was extracted. The transcriptome was sequenced by BioMaker Co. (Beijing, China). The unigenes annotated as *NAC* genes were subjected to ORF prediction (https://www.ncbi.nlm.nih.gov/orffinder/, accessed on 1 September 2022), the *NAC* gene family members with relatively complete ORFs were identified, and the corresponding aa sequences were obtained. The conserved functional domains of the putative proteins were predicted using tools at the NCBI (https://www.ncbi.nlm.nih.gov/cdd (accessed on 1 September 2022), and proteins without NAC functional domains were removed. The predicted aa sequences of the NAC transcription factors were analyzed using ProtParam (https://web.expasy.org/protparam/ (accessed on 1 September 2022)) and ProtComp 9.0 (http://www.softberry.com/berry.phtml?topic=pcompb&group=help&subgroup=proloc (accessed on 1 September 2022)) to determine their physicochemical properties and predicted subcellular location.

### 2.2. Motif, Phylogenetic Tree, and Protein–Protein Interaction Analyses

MEME (http://meme-suite.org/ (accessed on 1 September 2022) was used to analyze the conserved motifs of the *S. glauca* NAC family members. ClustalX2 software was used for aa sequence alignment, and the alignment results were used to construct a phylogenetic tree using MEGA7.0. To further explore the relationships among NAC proteins, a protein–protein interaction network was constructed based on the orthologs of SgNACs in *Arabidopsis*. The functional relationships of NAC proteins were predicted using the STRING protein interaction database (https://string-db.org/ (accessed on 1 September 2022)) (STRING 11.5), which predicts protein interactions on the basis of experimental data, computational analyses, and information in the literature. Gene Ontology (GO) classification analysis was performed for the NAC proteins using STRING 11.5.

### 2.3. Expression Profile Analysis of NAC Genes

Based on the transcriptome sequencing results of *S. glauca*, a gene transcript heatmap was drawn using Heatmap software (https://www.omicshare.com/tools/Home/Soft/heatmap (accessed on 1 September 2022)) on the Gedi Bio Cloud platform.

### 2.4. Subcellular Localization

Specific primers were designed to clone the *SgNAC6* and *SgNAC18* ORF sequences using the *S. glauca* cDNA as the template. The ORFs were each inserted into the plant expression vector pCAMBIA1302 (containing the gene encoding green fluorescent protein). Subsequently, each vector was transferred into onion epidermis via *A. tumefaciens* EHA105. The method was as follows: *A. tumefaciens* was propagated at a ratio of 1:100, cultured overnight, and centrifuged, and the cells were resuspended in a medium containing 10 mmol·L^−1^ MgCl_2_ and 100 μmol·L^−1^ acetyl-syringone. The inner epidermis of the onion was cut into small pieces, transferred to a solid medium, and cultured in the dark for 1 day. After co-culturing with the resuspended *A. tumefaciens* cells for 20 min, the bacterial solution was blotted from the surface of the onion epidermis with filter paper. The onion epidermis was then placed on solid MS medium and cultured in the dark for 1–2 days before observing the inner cells under a fluorescence microscope.

### 2.5. qRT-PCR Verification

The transcript levels of *SgNAC2*, *SgNAC14*, *SgNAC16*, and *SgNAC25* in the leaves of *S. glauca* after salt treatment were verified by qRT-PCR. The experimental materials used for these analyses were the same as those used for the transcriptome sequencing analyses, but the three replicates in the same treatment were pooled. The primers were designed using Primer 5 (Table 1). The PCR reagents were SYBR Green Real-time PCR Master Mix (TOYOBO, Tokyo, Japan), and the operating system was the LightCycler^®^ 480 Real-Time PCR System (Roche, Basel, Switzerland). The experiments were conducted with three replicates.

## 3. Results

### 3.1. Identification and Physicochemical Properties of the NAC Transcription Factor Family in S. glauca

We identified 43 unigenes with NAC annotations from the transcriptome data of *S. glauca* and obtained sequences for 25 NAC family members with relatively complete structural domains and open reading frames (ORFs) using the ORF prediction and CDD (Conserved Domain Database) conserved motifs tools at the NCBI website. The 25 *S. glauca* genes were named *SgNAC1–SgNAC25*. Then, we analyzed the physicochemical properties of the putative proteins based on their predicted amino acid (aa) sequences. The aa lengths of the *S. glauca* NAC transcription factors ranged from 103 (SgNAC21) to 476 (SgNAC1). The protein molecular weight ranged from 11,972.49 to 53,159.54 Da, and the theoretical isoelectric point ranged from 4.12 to 10.11. The subcellular localization analysis predicted that five NAC transcription factors localized outside the cell, and the remaining 20 NAC transcription factors localized in the nucleus (Table 2).

### 3.2. Evolutionary Analysis of NAC Families in S. glauca and A. thaliana

**Figure 1:**
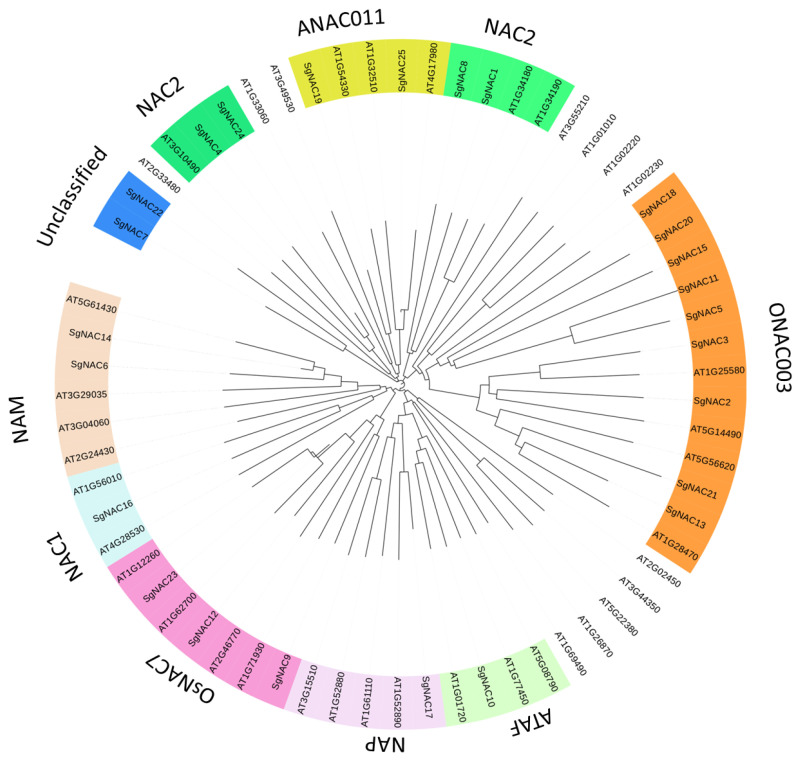
Phylogenetic relationships and subgroup designations of NAC transcription factors of *S. glauca* and *A. thaliana*.

An evolutionary analysis was conducted for the *S. glauca* NAC transcription factors alongside those of the model plant *A. thaliana*. In this analysis, 23 *S. glauca* NAC transcription factors were grouped in nine known NAC subfamilies (classified according to the *A. thaliana* NAC transcription factor family), and two *S. glauca* NAC transcription factors that were more distantly related to *A. thaliana* NAC transcription factors were not classified into any known *A. thaliana* subfamilies (Figure 1). Among the 23 *S. glauca* NAC transcription factors in known families, nine belonged to the ONAC003 subfamily, four belonged to the NAC2 subfamily, three belonged to the OsNAC7 subfamily, two belonged to the ANAC011 subfamily, two belonged to the NAM subfamily, and one belonged to each of the ATAF, NAP, and NAC1 subfamilies.

### 3.3. Conserved Motifs of NAC Family Transcription Factors in S. glauca

**Figure 2:**
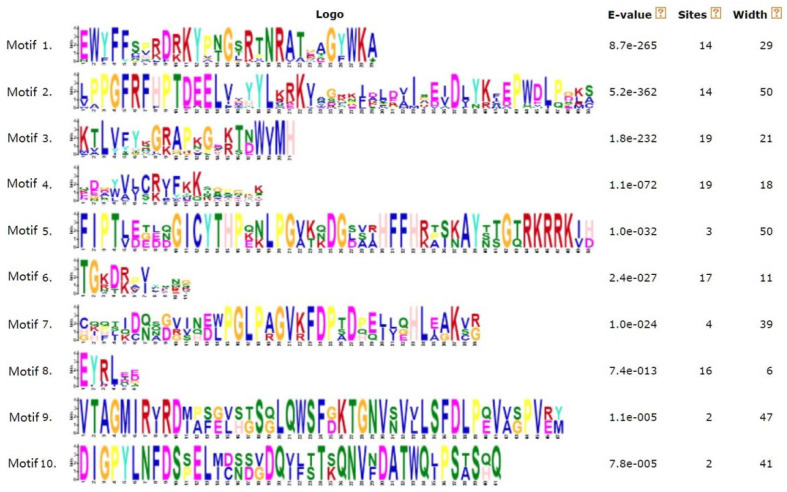
Identified motifs in NAC transcription factors of *S. glauca*.

**Figure 3:**
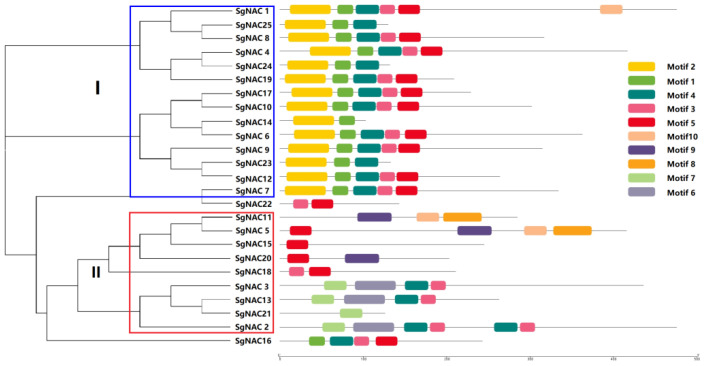
Distribution of identified motifs in NAC transcription factors of *S. glauca.* Blue indicated categories I and red indicated categories II.

We constructed an evolutionary tree for the 25 SgNAC amino acid sequences using the neighbor-joining method and used MEME online software version 5.5.2 to predict conserved motifs in the putative *S. glauca* NAC proteins. Ten conserved motifs were predicted (Figure 2). Based on the evolutionary relationships and distribution of the motifs, the *S. glauca* NAC family transcription factors were divided into two categories: I and II (Figure 3). Category I contained 14 members with conserved motifs 1 and 2. All but one of the category I NACs also contained one or more of conserved motifs 3–5. Category II contained nine members with one or more of conserved motifs 4–10. SgNAC16 and SgNAC22 were not included in these two categories; however, judging from the number and structure of their motifs, SgNAC16 lacked motif 2, and SgNAC22 lacked several conserved motifs among motifs 1–4. SgNAC14 and SgNAC15, which belonged to categories I and II, also lacked many of the conserved motifs.

### 3.4. Expression Analysis of NAC Genes in S. glauca

**Figure 4:**
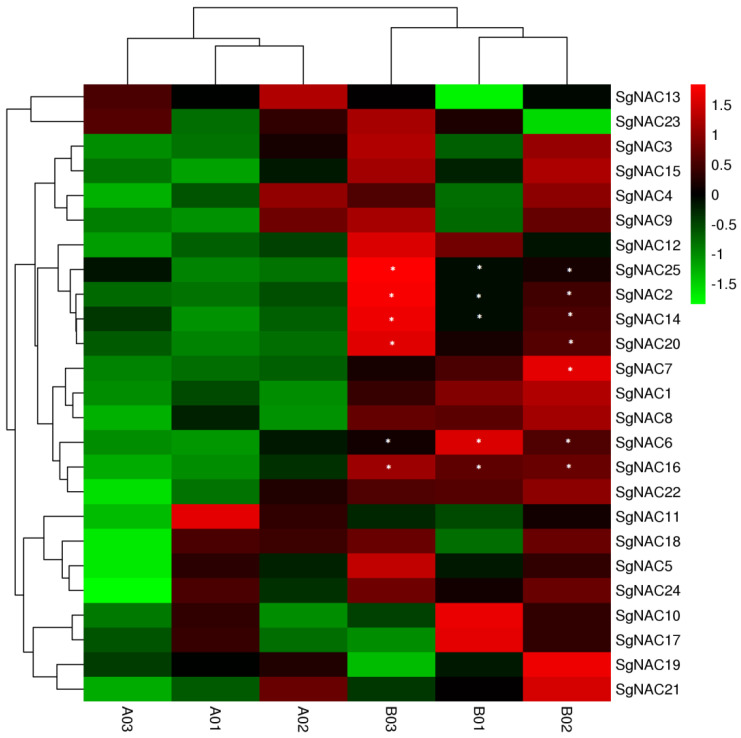
Transcript profiles of *NAC* genes in *S. glauca* under salt stress. A01–A03, water control (CK); B01–B03, 300 mmol·L^−1^ NaCl; * significant difference.

Based on the transcriptome sequencing data of *S. glauca*, we plotted an expression heatmap of the 25 NAC genes under 300 mmol·L^−1^ NaCl stress (B01–B03) and water as the control (A01–A03) (Figure 4). As shown in the figure, most *S. glauca NAC* genes were upregulated to some degree after 24 h of salt stress, and seven showed significant changes in their transcript levels.

### 3.5. Analysis of NAC Protein–Protein Interaction Network

**Figure 5:**
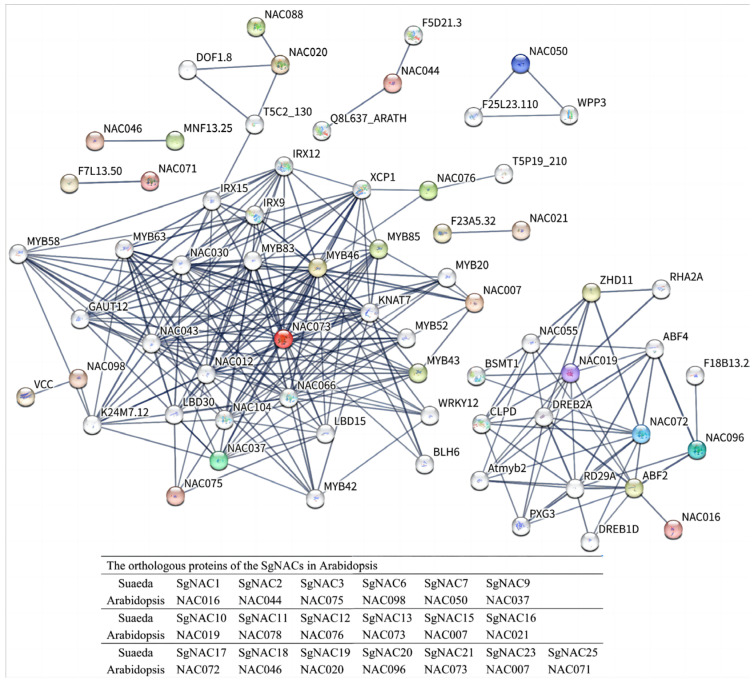
Protein–protein interaction network of SgNAC proteins based on their orthologs in *Arabidopsis*. Nodes represent proteins. Connections between nodes represent interactions between proteins, with edge thickness indicating the confidence level of the interaction. One-to-one correspondence of orthologous proteins between *Suaeda* and *Arabidopsis* is indicated below the figure.

Protein–protein interaction networks provide information about the regulatory networks among biomolecules and can be useful for predicting the functions of unknown proteins. In this study, the potential interactions among SgNAC proteins were explored using the STRING program based on the *A. thaliana* association model (Figure 5). The potentially interacting proteins were subjected to a Gene Ontology (GO) classification analysis. The proteins in the interaction network were mainly enriched in the following GO categories: positive regulation of the macromolecule metabolic process (GO:0010604), cell wall organization or biogenesis (GO:0071554), regulation of secondary cell wall biogenesis (GO:2000652), response to an oxygen-containing compound (GO:1901700), response to an abiotic stimulus (GO:0009628), response to a hormone (GO:0009725), and response to abscisic acid (GO:0009737).

### 3.6. qRT-PCR Verification of Transcript Profiles

**Figure 6:**
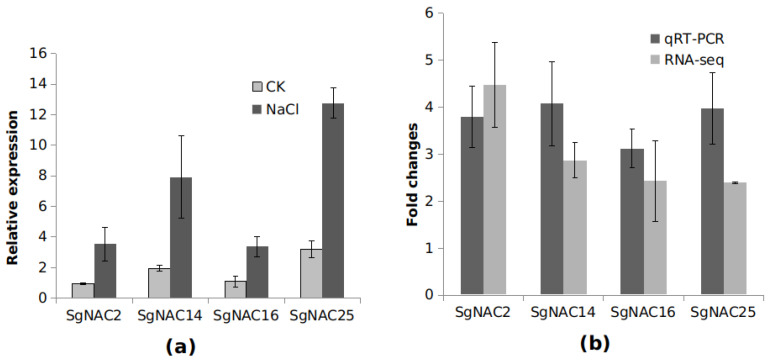
qRT-PCR confirmation of gene transcript levels in leaves of *S. glauca*. (**a**) qRT-PCR analyses of gene transcript levels in leaves in *S. glauca* after salt treatment and in the control (CK); (**b**) fold change in gene transcript levels as detected by RNA-Seq and qRT-PCR.

The transcript levels of *SgNAC2, SgNAC14, SgNAC16*, and *SgNAC25* in the leaves of *S. glauca* after salt treatment and in the control (CK) were verified with qRT-PCR. The results showed that, compared with the control, the salt treatment resulted in significant increases in the transcript levels of *SgNAC2*, *SgNAC14*, *SgNAC16*, and *SgNAC25* (Figure 6a). This profile was consistent with the trend detected from the RNA-seq data (Figure 6b), further verifying the reliability of the RNA-seq data.

### 3.7. Subcellular Localization of SgNAC6 and SgNAC18 Proteins

**Figure 7:**
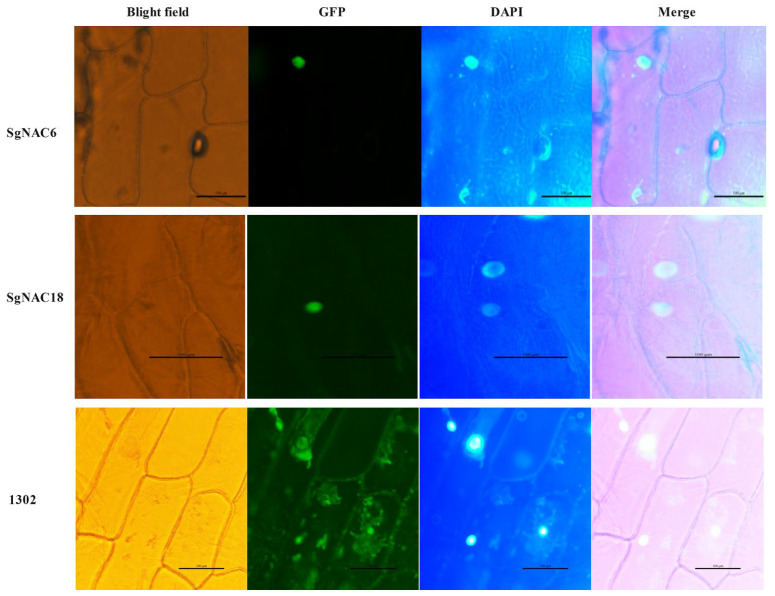
Sub-cellular localization of SgNAC6 and SgNAC18 in onion epidermal cells.

The plant expression vectors SgNAC6-1302 and SgNAC18-1302, which contained the coding sequences of the *SgNAC6* and *SgNAC18* genes, respectively, as well as the 1302 empty vector, were transduced into onion epidermis via *Agrobacterium tumefaciens.* The transformed cells were observed under a fluorescence inverted microscope. As shown in Figure 7, the cells harboring the 1302 empty vector showed green fluorescence in the cell membrane, cytoplasm, nucleus, and other parts. The cells were stained with DAPI as a nuclear marker. In onion epidermal cells expressing SgNAC6-1302 and SgNAC18-1302, green fluorescence signals were detected in the cell nucleus (in the same location as DAPI signals), indicating that the SgNAC6 and SgNAC18 proteins localized in the nucleus.

## 4. Discussion

The localization of the putative NAC proteins was predicted on the basis of their aa sequence. Most of the *S. glauca* NAC transcription factors were predicted to localize to the nucleus, and a few were predicted to localize in the extracellular space, similar to the localization patterns of other plant NAC transcription factors [17,18,19]. In the conserved motif analysis, the *S. glauca* NAC family transcription factors were divided into two groups according to their conserved motifs, and the motifs within each group were relatively conserved and consistent. Some *S. glauca* NAC transcription factors lacked certain conserved motifs. The evolutionary analysis with *A. thaliana* NAC transcription factor proteins showed that, except for SgNAC7 and SgNAC22, the remaining *S. glauca* NAC transcription factors belonged to nine known NAC subfamilies. These results suggest that the functions of NAC transcription factors are relatively conserved among different plant species.

Analyses of gene transcript profiles can provide information about gene function. After normalizing the transcript abundance data of the 25 *NAC* genes in *S. glauca*, it became clear that most of the *NAC* genes were upregulated to some degree by salt stress (Figure 4). The upregulation of these genes under salt stress suggests that they may be involved in the stress response, as well as in plant growth and development. The proteins predicted to interact with NAC proteins (Figure 5) were mainly enriched in the GO terms of cell wall organization or biogenesis (GO:0071554), response to an abiotic stimulus (GO:0009628), and response to a hormone (GO:0009725), consistent with the transcript profiles of *SgNACs* under salt stress.

Previous studies on the classification and function of NAC transcription factors have shown that genes with similar sequences in the same evolutionary groups often have similar protein functions. Therefore, we conducted a functional prediction analysis of the *NAC* genes in *S. glauca*. Soybean *GmNAC5*, which belongs to the NAM subfamily, is induced by high salt and other abiotic stresses [20]; *SgNAC6* and *SgNAC14* genes, which cluster in the NAM subfamily, are induced by salt stress, and their encoded products may play roles in responses to salt stress and plant hormones. *Arabidopsis* AtNAP [21] plays a negative regulatory role in the salt stress response by inhibiting the expression of *AREB1*; *SgNAC7*, which clusters in the NAP subfamily, is induced by salt stress and may also have functions in the responses to salt stress and plant hormones. OsSNAC1 and OsNAC3 in rice and TaNAC2 in wheat belong to the ONAC003 subfamily. Overexpression of *OsSNAC1* in transgenic rice was shown to increase resistance to drought and salt stresses [22], and *OsNAC3* was shown to respond to multiple environmental and hormone signals [23]. Overexpression of *TaNAC2* in *A. thaliana* [10] led to improved resistance to multiple abiotic stresses. *SgNAC2* and *SgNAC20*, which also cluster in the ONAC003 subfamily, are induced by salt stress and may play important roles in the apadtation to the alkali environment of *S. glauca*. The *A. thaliana* NAC2 subfamily member, ANAC016, can activate abscisic acid signaling and improve drought resistance. Whether *SgNAC1* and *SgNAC8* in the NAC2 subfamily have similar functions is yet to be determined. Further research is required to explore the specific functions of the NAC transcription factors in *S. glauca*. Based on the comprehensive analysis in this study, we concluded that *S. glauca NAC* genes play a role in the salt stress response of *S. glauca*, but further research is required to explore their mechanisms of action.

The results of previous studies [10,22,23] have shown that NAC transcription factors play important roles in the salt stress response through different regulatory pathways. Based on analyses of the transcriptome data of *S. glauca* under salt stress, we have identified 25 *NAC* genes and predicted the structure, function, and subcellular localization of their encoded products. These results lay the foundation for further verification of their roles and functions. Together, our results indicate that some *S. glauca* NAC transcription factors are involved in the response to salt stress. This adds to the current state of our knowledge about the involvement of NAC transcription factors in plant resistance, especially in halophytes.

## Figures and Tables

**Table 1:** Sequences of primers used in this study.

Gene	Primer Sequences
*SgNAC2*	For-CCGAACTGCAAGTTTTCGAT
*SgNAC2*	Rev-GGGTGTAGCAAATCCCTTGA
*SgNAC14*	For-GCCACCATATCCCTCTTCAA
*SgNAC14*	Rev-CAAATCCCATTTTCACCTTCA
*SgNAC16*	For-TCGGATTGAAGGACCTTTTG
*SgNAC16*	Rev-AGTCCATCAATGGCGGTAAG
*SgNAC25*	For-CGCGATATGGAGTGGTTCTT
*SgNAC25*	Rev-TCATGCATCACCCAATCAGT
*Actin*	For-CCGCAAAGATTACATACC
*Actin*	Rev-TCACCGAAAGTGCTTCTA

**Table 2:** Physicochemical properties of putative NAC transcription factors of *S. glauca*.

Name	Number of Amino Acids	Molecular Weight	Theoretical pI	Subcellular Location
SgNAC1	476	53,159.54	5.67	Extracellular
SgNAC2	476	53,734.52	5.45	Nuclear
SgNAC3	436	48,661.99	4.80	Nuclear
SgNAC4	417	46,272.22	5.30	Nuclear
SgNAC5	416	45,552.67	4.29	Extracellular
SgNAC6	363	40,803.62	6.07	Nuclear
SgNAC7	334	38,096.55	8.08	Nuclear
SgNAC8	317	35,238.61	4.85	Nuclear
SgNAC9	315	36,363.36	6.59	Nuclear
SgNAC10	302	34,461.15	7.04	Nuclear
SgNAC11	285	30,799.18	4.12	Extracellular
SgNAC12	264	30,666.56	8.22	Nuclear
SgNAC13	263	29,969.94	9.03	Nuclear
SgNAC14	126	13,603.23	6.00	Nuclear
SgNAC15	245	28,377.47	6.01	Nuclear
SgNAC16	243	27,807.89	7.70	Nuclear
SgNAC17	229	26,021.46	9.55	Nuclear
SgNAC18	211	24,039.92	5.75	Nuclear
SgNAC19	209	24,255.47	9.02	Nuclear
SgNAC20	203	22,706.82	4.67	Extracellular
SgNAC21	103	11,972.49	5.35	Nuclear
SgNAC22	143	16,120.71	4.59	Extracellular
SgNAC23	133	15,816.1	9.21	Nuclear
SgNAC24	132	15,558.77	10.11	Nuclear
SgNAC25	130	15,335.51	8.85	Nuclear

## Data Availability

Data were uploaded to the NCBI database under the accession number PRJNA295637 (https://www.ncbi.nlm.nih.gov/bioproject/PRJNA295637/ (assessed on 15 September 2015).
